# Supplementary material for: Acceptability of tongue swabs for tuberculosis screening in migrant settings in northern Italy: A qualitative study
Source: PLOS Glob Public Health. 2026 Mar 6;6(3):e0004908. doi: 10.1371/journal.pgph.0004908 (PMC12965601; doi:10.1371/journal.pgph.0004908)
Supplement: S1 Text — (DOCX) [file pgph.0004908.s001.docx]

**Semi-structured interview guide for interviews with migrants:**

**User Acceptance of Migrants Using Tongue Swabs for TB Sample Collection**

[**https://www.youtube.com/watch?v=e4n4FdAh7UY&list=LL&index=2&ab_channel=UWDEOHS**](https://www.youtube.com/watch?v=e4n4FdAh7UY&list=LL&index=2&ab_channel=UWDEOHS)

**Introduction:**

My name is [name], and I am a [position] at the [institution] in [city, country]. This interview is part of my [study]. I [state positionality to participants]. I understand your desire to flee your country and find refuge in Europe. My experiences shaped me in many ways and fueled my passion to help migrants access better healthcare.

I am grateful that you are here. I want to learn more about your experience with the healthcare system in Italy so that we can improve it for others who are coming. Your participation will make seeking care a better experience for you as you continue and for others in the future. Taking care of your health is the most important thing you can do for yourself and your loved ones. Then you can concentrate on what you need to do to adapt and create a new life here in Europe.

**Explanation of the process**

If you agree to participate in this study, we will be asking you questions to understand what has helped or hindered you from accessing health care during your time here in Italy. We will ask you questions regarding your migration journey to understand how that has impacted your health and what support you need. We will also go through a simulation and ask for your preference for 2 types of tuberculosis diagnostic tests.

**Note**:

- In-depth interview time: 90 minutes
- Focus groups duration 2 hours.

We will be taking notes and audio recording the sessions. The recordings help us to make sure we can be present with you in the conversation and allow us to review information later. Audio recordings will be professionally transcribed for data analysis. Once transcribed, we will destroy your audio recording so your voice and name will not be associated with the transcribed text. Your name or contact information will not be associated with any data that is shared in such a repository or with other researchers.

The findings may be written into a report or manuscript for publication in a peer-reviewed journal. We will not refer to you by name in any report or publication. Any identifying data will not be available to the public.

**Does this make sense?**

Your participation in this session is completely voluntary, and you may opt to stop at any point in the interview process and leave the study at any time. You will not be penalized for not answering questions or leaving the study. We will provide you with a link to the complete copy of our results after the study.

**Does this make sense?**

**If you want more information,** you can talk to the study team. Luigi Ruffo Codecasa is the lead researcher at Niguardia Hospital for this study and can be contacted at +39 xxxxxxxxx. You may also contact Gabriella Parini at [share contact].

If you want to talk with someone who is not part of the study team about the study, your rights as a research participant, or to report problems or complaints about the study, all contacts are available in the informed consent sheet you just signed. You may also contact the UW Human Subjects Division at (206) xxx-xxxx or [share contact].

Access to your information will be limited to certain members of the research team and individuals from the UW who may need to audit study records.

**Questions and consent**

Do you have any questions before we begin?

Do you agree to be a participant in our study? (Ask participants to provide a verbal “yes”.)

Do you agree to the audio recording of this interview? “If not, then we need an additional person as a scribe and focus on key quotes.”

**Demographic info part A:**

**Intro Demographic Information:**

1. Before we continue, how would you like me to refer to you? Ask for spelling if needed.
2. What is your gender identification? Would you say you are a male, or female, or something else?
3. What is your ethnicity?
4. What is your date of birth?
5. How many years of schooling do you have?
6. What language/s do you use at home?
7. Did you come from a rural or urban environment?
8. What community or tribe do you identify with? ( pilot with the cultural translator to see when appropriate for what community)
9. Religion? What is your religious community? Muslim? Christian? Spiritual? Agnostic? Etc
10. Marital status (single, divorced, widowed, poly?)
11. Do you have children? If so, how many? Note: phrase in a culturally sensitive manner as they may have lost children
12. What kind of permission are you seeking to stay in Italy? Asylum seeker, work permit, sanitoria (in queue for years and not processed yet), if female, family reunification.
13. Tell me about your migration journey.
    1. Where are you from?
    2. What country are you originating from?
    3. How did the journey impact your health (mental, physical)?

**Ice breaker: What are you happy about in your life? What brings you joy here in Italy? What are your dreams of creating a life here in Europe?**

**Questions:**

1. What brought you in today? Did you ask to come ( to the clinic or reception center), or are you here for a routine (check-up or class?)
2. What made it easy for you to come in and speak with me?
3. What would help you to come in and seek medical care when you are not feeling well or need a routine check-up?
   1. What would support you?
4. What are the things that you need to make it easy to come into the clinic?
5. What are the consequences of your falling ill? How would getting sick impact your life here in Milano? In the welcome center (or wherever they live), your job is?

**Health care seeking behaviors:**

1. How did you make sure you and your family were taken care of where you come from?
   1. In ( insert hometown), where would you go when you were sick? Who would you trust to treat you and your family? Is there anyone or an institution that you would not trust in seeking care?
2. How is that different here in Italy?
3. What makes it hard for you to come into the clinic?
4. What would make it easier?
5. When you wake up in the morning, do you cough?

**Baseline of TB background and diagnosis**

1. What do you know about TB?
   1. If no…It is a respiratory disease that causes cough, fever, night sweats, and severe weight loss. It is the leading cause of death in the world.
   2. Have you heard of other respiratory diseases like COVID-19 or the flu?
2. If yes… what do you know about TB?
3. Have you been tested for TB before?
4. Did you have family members who had a cough or night sweats, or had trouble holding weight?

**Oral hygiene questions**

1. Are you taking any medication? If so, what?
2. Did you eat breakfast?
3. Did you brush your teeth this morning?
4. When you brush your teeth, do you notice your gums bleeding?
5. Do you use dental floss?
6. Do you clean your tongue?
7. Do you use mouthwash?
8. When was the last time you went to the dentist?
9. Are you a smoker, tobacco, e-cigarette, or other user? If so, how often? How many packs a day?

**Ways to test for TB**

There are several different ways to test for TB, such as the blood test and chest x-ray. The tuberculin skin test will tell us if you had any kind of response to the vaccine. The blood test is an antibody test that can inform us if you have a specific differentiation from someone who has just had the vaccine versus someone who has latent TB. It can not tell us the disease progression, so we don't know if it is latent vs active.

**Questions related to the evaluation of the educational resources on how to self-swab:**

**Show a video on how to self-swab in the waiting room before the interview starts, or should we show the video live with the participant there?**

**Link to Swab collection procedures: Reference images from the SSS protocol.**

**Supervised Self-Swab Sample Collection Procedures for TB screening: illustration and instructions**

Step 1: Carefully **open the swab wrapper** at the easy-peel stick end. Remove the swab from the base of the handle. Avoid touching the swab head.

Step 2: Open your mouth and **stick out your tongue**.

Step 3: Firmly press the swab to the **top of your tongue** and move it **right to left and up and down**. Rotate the swab in your fingers as you go.

Step 4: **Swab your tongue for 15 seconds**. Be careful to only swab as far back as is comfortable.

Step 5: **Open the container** for the swab and **insert** it with the **swab head down**. You will see a small notch in the handle of the swab. This is the breakpoint. **Push the breakpoint against the inside edge of the tube and snap the stick.** Discard the remaining stick. With your swab inside the tube, screw the lid on tightly and hand it back to the health care provider

**Now collect a sputum sample: VERY Important to take the swab sample BEFORE the Sputum**

Take a very deep breath and hold the air for 5 seconds. Slowly breathe out. Take another deep breath and cough hard until some sputum (the viscous liquid from your lungs) comes up into your mouth. Spit the sputum into the plastic cup and put the lid on tightly. Please note: Sputum is material that is brought up from the lungs, and nasal secretions and saliva or spit are not the same.

1. Inhale deeply, as far as possible, and then exhale slowly three times.
2. After the third breath, inhale completely and try to cough hard to produce sputum from deep in the lungs. You may feel a rattle or tickle as the sputum moves up the lungs and into the throat.
3. Expectorate/spit the sputum into a sterile specimen container.
4. When there is at least 5 ml (1 teaspoon) of sputum, replace the lid on the container and tighten it so it does not leak.
5. Return it to me

**We would like to hear about your experience with providing samples for TB screening.**

1. Have you been tested for TB back home before?
2. Has anyone in your family been diagnosed with tuberculosis before?
3. Describe your experience with producing sputum here at the clinic.
   1. What made it easy?
   2. What made it difficult?
4. Describe your experience with the tongue swab sample collection method. Did you swab yourself, or did the nurse do it for you?
   1. What made it easy?
   2. What made it difficult?
5. Do you have a preference for either of them, and if so, then why?
6. What do you know about self-testing? COVID-19, pregnancy, ovulation, or HIV?
7. If yes, did you ever self-swab? Or did a healthcare worker do it?
8. What educational resources do you need to understand how to produce sputum more easily?
9. What education resources do you need to understand how to make tongue swabbing easier?
10. Are there any scenarios where you think you wouldn't like to produce sputum?
11. Are there any scenarios where you think you wouldn't like to provide a tongue swab?
12. What educational resources do you need to understand how to protect yourself and your community from tuberculosis disease in the future?

**Self Care**

1. How does dealing with people going through painful experiences impact you?
2. How do you recharge for your self-care? What brings you joy here in Italy?
3. What do your loved ones need to take care of themselves?

**Vaccines**

1. Do you have the TB vaccine?
2. What other vaccines do you have?
3. Are there other vaccines you want to get?

**Closing**

Thank you so much for sharing your expertise and experiences. Your perspective will guide us in creating solutions for an easier process of getting a TB diagnosis in the future.

1. I want to make sure that I understand your responses and I’m fully representing your experience. (Summarize their responses and ask for clarity.) Which test was harder?
   1. How can we make it easier for you to get the healthcare you need here in Italy?
2. Is there anything else you would like to share before we go?
3. Can we contact you if we have additional questions in the future?
4. Are you OK?
   1. Is there any other information you need?
   2. What other services could I connect you with?

You should be very proud of yourself for being here and all that you have endured. This is a temporary measure; you are taking the necessary steps to improve the quality of life for yourself and your loved ones. Thank you very much for your participation. Wishing you (and your family) well during this transition.
