# Supplementary material for: Acceptability of tongue swabs for tuberculosis screening in migrant settings in northern Italy: A qualitative study
Source: PLOS Glob Public Health. 2026 Mar 6;6(3):e0004908. doi: 10.1371/journal.pgph.0004908 (PMC12965601; doi:10.1371/journal.pgph.0004908)
Supplement: Attachment — Submitted filename: Response to Reviewers_Final.docx [file pgph.0004908.s003.docx]

October 10^th^, 2025

Miguel Angel Garcia-Bereguiain, PhD
Academic Editor
PLOS Global Public Health

Dear PLOS Editors,

On behalf of the authors, we would like to thank *PLOS Global Public Health* for your review of our manuscript. We welcome the opportunity to resubmit with revisions that respond to the reviewers’ comments and recommendations. We have carefully considered their reviews and provided responses to each point as well as relevant changes to the manuscript. Two versions of the manuscript are being resubmitted, including one clean and one with changes tracked in red.

Sincerely,

Daniela Maria Cirillo

**EDITORS COMMENTS**

*We express our gratitude to the Editors and Reviewers for their positive responses and insightful comments about our work. Their observations helped us to thoroughly revise and refine the manuscript. We hope that the revised manuscript has improved in terms of scientific value, presentation, and language, and is now suitable for publication.*

*Please find below our point-by-point response to the comments.*

**Journal Requirements:**

1. Please include a complete copy of PLOS’s questionnaire on inclusivity in global research in your revised manuscript. Our policy for research in this area aims to improve transparency in the reporting of research performed outside of the researchers’ own country or community. The policy applies to researchers who have travelled to a different country to conduct research, research with Indigenous populations or their lands, and research on cultural artefacts. The questionnaire can also be requested at the journal’s discretion for any other submissions, even if these conditions are not met. Please find more information on the policy and a link to download a blank copy of the questionnaire here: [https://journals.plos.org/globalpublichealth/s/best-practices-in-research-reporting](https://urldefense.com/v3/__https://journals.plos.org/globalpublichealth/s/best-practices-in-research-reporting__;!!K-Hz7m0Vt54!l0KPiz74t3avQOFz_7DXWJZGZtYB3h4jqvvVFCb4o8Kowdy82mfCcp6lV3pq0yIk9KUXFFU153WZapYvmAlfpuY$). Please upload a completed version of your questionnaire as Supporting Information when you resubmit your manuscript.

***Thank you for your question. We have completed the PLOS questionnaire on inclusivity in global health research and uploaded it under supporting information.***

2. Please provide a detailed online Financial Disclosure statement. This is published with the article. It must therefore be completed in full sentences and contain the exact wording you wish to be published.

a) State the initials, alongside each funding source, of each author to receive each grant, if applicable. For example: “This work was supported by the National Institutes of Health (####### to AM; ###### to CJ) and the National Science Foundation (###### to AM).”

b) State what role the funders took in the study. If the funders had no role in your study, please state: “The funders had no role in study design, data collection and analysis, decision to publish, or preparation of the manuscript.”

***We thank you for these comments. We have revised the manuscript and included the following information under the Financial Disclosure statement.***

***Financial disclosure statement:***

***This work was supported by the National Institute of Health grant U54EB027049 (RC and GAC) and by the University of Washington Office of the Provost (RC). The funders had no role in the study design, data collection and analysis, discussion to publish, or preparation of the manuscript.***

3. Please ensure that the funders and grant numbers match between the Financial Disclosure field and the Funding Information tab in your submission form. Note that the funders must be provided in the same order in both places as well.

***We thank you for this comment. The funders and grant numbers match between the Financial Disclosure field and the Funding Information tab in the submission form.***

4. Please update your online Competing Interests statement. If you have no competing interests to declare, please state:

***We have included in the manuscript and also the online Competing Interests statements to note that “The authors have declared that no competing interests exist.***”

5. In the online submission form, you indicated that “The data supporting the findings of this study are not publicly available but can be obtained from the corresponding author upon reasonable request.”.

All PLOS journals now require all data underlying the findings described in their manuscript to be freely available to other researchers, either
a) In a public repository,
b) Within the manuscript itself, or
c) Uploaded as supplementary information.

This policy applies to all data except where public deposition would breach compliance with the protocol approved by your research ethics board. If your data cannot be made publicly available for ethical or legal reasons (e.g., public availability would compromise patient privacy), please explain your reasons by return email, and your exemption request will be escalated to the editor for approval. Your exemption request will be handled independently and will not hold up the peer review process, but it will need to be resolved should your manuscript be accepted for publication. One of the Editorial team will then be in touch if there are any issues.

***Thank you for sharing this request. Due to the sensitivity of the topic and the participants, the IRB from the University of Washington in the USA, the University of San Raffaele, and Niguarda hospital in Italy approved the work with the condition that data would not be available for secondary analysis by anyone other than the study team, and that only limited members of the study team could access the data. Release of the data would require approval from the IRBs for additional analyses. As a result, we have an exemption for public availability of any data including memos, recordings, transcripts, or the Rapid Analysis Framework matrix. Public deposition of the Rapid Analysis Framework Matrix would breach compliance with the protocol approved by the University of Washington IRB research ethics board, as well as the Italian ethical board. Public Availability would compromise participant privacy. As a result, the data shall not reside in any sort of structured database or registry. We have provided Figure 2, which shows the categorization of the COM-B domains that were used to develop the matrix. Thank you in advance for reviewing our exemption request.***

6. We have noticed that you have uploaded Supporting Information files, but you have not included a list of legends. Please add a full list of legends for your Supporting Information files before or after the references list.

***Thank you for this comment. We have included in the manuscript and Supporting Information files the list of legends for***

***I- Semi-structured interview guide***

***II- Inclusivity in global research***

7. Some material included in your submission may be copyrighted. According to PLOS’s copyright policy, authors who use figures or other material (e.g., graphics, clipart, maps) from another author or copyright holder must demonstrate or obtain permission to publish this material under the Creative Commons Attribution 4.0 International (CC BY 4.0) License used by PLOS journals. Please closely review the details of PLOS’s copyright requirements here: PLOS Licenses and Copyright. If you need to request permissions from a copyright holder, you may use PLOS's Copyright Content Permission form.

Please respond directly to this email or email the journal office and provide any known details concerning your material's license terms and permissions required for reuse, even if you have not yet obtained copyright permissions or are unsure of your material's copyright compatibility.

Potential Copyright Issues:

Images in “Semi-structured interview guide_ migrants .docx”: Please confirm whether you drew the images / clip-art within the figure panels by hand. If you did not draw the images, please provide (a) a link to the source of the images or icons and their license/terms of use; or (b) written permission from the copyright holder to publish the images or icons under our CC-BY 4.0 license. Alternatively, you may replace the images with open source alternatives. See these open source resources you may use to replace images / clip-art:
- [https://commons.wikimedia.org](https://urldefense.com/v3/__https://commons.wikimedia.org__;!!K-Hz7m0Vt54!l0KPiz74t3avQOFz_7DXWJZGZtYB3h4jqvvVFCb4o8Kowdy82mfCcp6lV3pq0yIk9KUXFFU153WZapYvG8kicpQ$)
- [https://openclipart.org/](https://urldefense.com/v3/__https://openclipart.org/__;!!K-Hz7m0Vt54!l0KPiz74t3avQOFz_7DXWJZGZtYB3h4jqvvVFCb4o8Kowdy82mfCcp6lV3pq0yIk9KUXFFU153WZapYvsBPvQGc$)

***We appreciate your comment. All the images and figures were hand-designed by the study team. The images in the interview guide have been removed.***

8. We noticed that you used “Unpublished master’s thesis” in the manuscript. We do not allow these references, as the PLOS data access policy requires that all data be either published with the manuscript or made available in a publicly accessible database. Please amend the supplementary material to include the referenced data or remove the references.

***We appreciate your comment and your sharing the PLOS data access policy, which states that master's theses are not allowed in citations. We have removed this citation and replaced it with one published by PLOS Global Public Health.***

9. We do not publish any copyright or trademark symbols that usually accompany proprietary names, eg (R), (C), or TM (e.g. next to drug or reagent names). Please remove all instances of trademark/copyright symbols throughout the text, including ® on page 20.

***Thank you for sharing this publishing requirement. We have removed all copyright and trademark symbols in the manuscript.***

10. If the reviewer comments include a recommendation to cite specific previously published works, please review and evaluate these publications to determine whether they are relevant and should be cited. There is no requirement to cite these works unless the editor has indicated otherwise.

***Thank you for this reminder. The reviewers did not include a recommendation to cite specific previously published works.***

11. Please review your reference list to ensure that it is complete and correct. If you have cited papers that have been retracted, please include the rationale for doing so in the manuscript text, or remove these references and replace them with relevant current references. Any changes to the reference list should be mentioned in the rebuttal letter that accompanies your revised manuscript. If you need to cite a retracted article, indicate the article’s retracted status in the References list and also include a citation and full reference for the retraction notice.

***Thank you for this comment. Five additions to the reference list have been made to support the clarifying statements requested by the Reviewers.***

***17.*** [***Bezuidenhout, C. et al. Using sputum and tongue swab specimens for in-home point-of-care Targeted Universal testing for Tb of household contacts: An acceptability and feasibility analysis. medRxiv (2024) doi:***](http://paperpile.com/b/MfAM48/xKgz)[***10.1101/2024.11.01.24316570***](http://dx.doi.org/10.1101/2024.11.01.24316570)[***.***](http://paperpile.com/b/MfAM48/xKgz)

***35.*** [***Hamilton, A. B. & Finley, E. P. Qualitative methods in implementation research: An introduction. Psychiatry Res. 280, 112516 (2019).***](http://paperpile.com/b/MfAM48/j8XL)

***56.*** [***Wood, R. C. et al. Characterization of oral swab samples for diagnosis of pulmonary tuberculosis. PLoS One 16, e0251422 (2021).***](http://paperpile.com/b/MfAM48/jdJ1)

***57.*** [***Church, E. C. et al. Oral swabs with a rapid molecular diagnostic test for pulmonary tuberculosis in adults and children: a systematic review. Lancet Glob. Health 12, e45–e54 (2024).***](http://paperpile.com/b/MfAM48/Qgai)

***58.*** [***Martinson, N. A. et al. Evaluating systematic targeted universal testing for tuberculosis in primary care clinics of South Africa: A cluster-randomized trial (The TUTT Trial). PLoS Med. 20, e1004237 (2023).***](http://paperpile.com/b/MfAM48/Vej2)

***In addition, one reference (21) was replaced to amend a typo in the last name referenced (de Nooy as opposed to Nooy de.)***

***Moreover, we converted Olson et al (Ref 8) from a preprint to a published study, as well as Rockman et al (Ref 16).***

**REVIEWERS COMMENTS**

**Reviewer #1:**

I believe this paper represents a valuable contribution to the literature on TB diagnosis and migrant health from a qualitative perspective. Overall, it is well written and substantiated, highlighting supervised self-swabbing (SSS) and the use of the COM-B model as strengths. This approach could even be applied to other respiratory infections where current sample collection methods are invasive or impractical.

***We thank the Reviewer for the positive comments and review of our manuscript.***

I have the following minor comments:

- Only 24 interviews were conducted, with a strong gender imbalance (19 men, 5 women). This limits generalizability, particularly given potential gendered differences in health-seeking behavior. Please mention this in the discussion paragraph where the limitations of the study are discussed.

***Thank you for the comment. Qualitative studies seek and prioritize depth of information versus quantity. Line 196 shares our definition of, “Saturation as the point where no new themes emerged from additional interviews.” Our sample was sufficient to achieve saturation of several themes across interviews. Nevertheless, we agree with the reviewer that the sample size might impact generalizability and have included it as a limitation in the Discussion section lines 436-438.***

***“Despite the underrepresentation of women in the study, our sample size was sufficient to achieve saturation of several themes across interviews. This notwithstanding, our findings may not be generalizable to women in other settings.”***

- Recruitment challenges with migrants from West Asia and North Africa are acknowledged but deserve further elaboration. How might this underrepresentation bias results?

***We appreciate this important observation. We have now elaborated (lines 441-446) in the limitations section that the underrepresentation of participants from Southwest Asia and North Africa may have introduced bias, as their perspectives and experiences might differ from those of the groups strongly represented in our study. This underrepresentation may have limited the breadth of cultural views captured, particularly regarding attitudes towards screening and sample provision. To help address this, we have highlighted the need for future studies to use tailored recruitment strategies and culturally appropriate support to ensure that these key populations are more fully represented.***

***“Future studies need to intentionally design recruitment strategies to support hard to reach demographics, such as females and participants from Pakistan and the Southwest Asia and North Africa region to understand their perspectives, as they represent a significant portion of the migrant population in Italy who are underserved. This underrepresentation could influence the generalizability of the findings, as the perspectives of women and men from these groups could differ from those represented in the study.”***

- While rapid qualitative analysis is justified, a deeper description of how disagreements were resolved during coding would strengthen transparency.

***Thank you for the comment. We used a deductive framework-based method (Hamilton’s rapid qualitative analysis method) that does not use coding, but rather a matrix analysis. This reference is in line 176. We have clarified the process (lines 178-182).***

***“First, rapid data extraction and categorization were conducted by each researcher, entering their interview notes into a structured research matrix according to Hamilton's rapid qualitative analysis method. The matrix categorization was primarily based on the discussion guides and the thematic concepts of interest. Each transcript was reviewed by 2 people (RC and FS) and no discrepancies were identified between coders. A second analyst then cross-checked the data by listening to the audio recordings and refining the matrix.”***

***We also clarified that the interpretation of thematic analyses was discussed among reviewers to obtain a consensus, as now detailed (line 188-189)****.*

***“Interpretation of thematic analyses was discussed among reviewers to obtain a consensus.”***

- The discussion sometimes overstates the potential of SSS without equally weighing concerns about its diagnostic sensitivity compared to sputum. Although the focus is on acceptability, readers may expect a more balanced perspective on clinical utility.

***Thank you for the comment. We have added to the discussion section lines 414-422 that address swab utility beyond the clinic and TUTT based screening approaches. Within that we also now explicitly acknowledge the lower sensitivity and mention potential adoption resistance. Supporting references were also added as needed (Ref 56,57,58).***

***“Outside of the study participants’ views on TS, the fact that TS have shown lower sensitivities than sputum***[***^8,12,56,57^***](https://paperpile.com/c/MfAM48/PGSoj+jdJ1+Qgai+Lc9uW) ***may hinder its adoption in some settings. However, the use of TS could increase diagnostic yield because TS collection is not incumbent on sputum production. The importance of diagnostic yield has been emphasized recently, particularly in at-risk populations like migrants who may not be able to expectorate sputum.18 TB screening in a Targeted Universal TB testing (TUTT) approach, where people are screened based on risk rather than symptom presentation or care-seeking behaviors, would benefit from non-sputum samples like TS.^58^ TUTT is an example of a use case where lower sensitivity may be tolerated in favor of potentially higher diagnostic yields.^21^”***

- It would be helpful to more explicitly address whether findings are generalizable beyond Northern Italy, especially to higher TB burden or lower-resource migrant-receiving settings.

***We have clarified in the text that while the findings may not be fully generalisable, they provide useful insights for similar European migrant centres, with caution needed when extrapolating to lower-resource or higher TB burden settings (lines 450-455).***

***“As this was a single-centre study with predominantly male participants, the findings may not capture the full diversity of migrant experiences, particularly those of women, and may not be generalisable to different settings, particularly those that have fewer resources and a higher TB burden. Nonetheless, the themes identified provide useful insights that can inform future research and programme development in European migrant centres.”***

- Improve graphs quality (figures 1 and 2). These should not be pixelated.

***Thank you. Figures 1 and 2 have now been uploaded of high quality to ensure they are not pixelated.***

**Reviewer #2:**

The topic is highly relevant and innovative, as it explores tongue swabs as a practical alternative to sputum collection, particularly in migrant settings.

***We thank the Reviewer for their positive feedback. Please find below our point-by-point response.***

Minor Concerns:
1. It is important to mention that, as the study was conducted in a single referral center, the findings may not be fully generalizable to other migration contexts in Italy or Europe.

***Thank you for the comment. This is an important limitation of the study, and it is now discussed in more depth in the relevant Discussion paragraph at lines 450-455.***

***“As this was a single-centre study with predominantly male participants, the findings may not capture the full diversity of migrant experiences, particularly those of women, and may not be generalisable to different settings, particularly those that have fewer resources and a higher TB burden. Nonetheless, the themes identified provide useful insights that can inform future research and programme development in European migrant centres.”***

2. The conclusion could be strengthened with a clearer call for public policies that support the integration of multilingual, culturally adapted audiovisual materials into TB screening programs.

***Thank you for this valuable comment. We have revised the conclusion to include a clearer call for public policies that support the integration of multilingual, culturally adapted audiovisual materials into TB screening programmes at lines 463-466.***

***“These findings support the need for the development of public policies and health promotion materials that integrate multilingual, culturally adapted audiovisual materials into TB screening programs to enhance accessibility, engagement, and uptake among diverse migrant populations.”***
